# Data on the evaluation of the relation between β-arrestin 2 and YAP phosphorylation in patient-derived colon cancer organoids

**DOI:** 10.1016/j.dib.2022.108506

**Published:** 2022-08-02

**Authors:** Minsuh Kim, Ji Min Kim, Eun Jeong Cho, Chang Ohk Sung, Joon Kim, Se Jin Jang

**Affiliations:** a Asan Institute for Life Sciences, Asan Medical Center, Seoul, Republic of Korea; b Department of Pathology, Asan Center for Cancer Genome Discovery, Asan Medical Center, University of Ulsan College of Medicine, Seoul, Republic of Korea; c University of Ulsan College of Medicine, Seoul, Republic of Korea; d Graduate School of Medical Science and Engineering, KAIST, Daejeon, Republic of Korea

**Keywords:** β-arrestin 2, Yes-associated protein (YAP), Hippo pathway, Cancer organoid, Patient-derived model

## Abstract

The data presented in this article is related to a rapid communication entitled “β-arrestin 2 suppresses the activation of YAP by promoting LATS kinase activity”. This article describes the correlation of β-arrestin 2 and YAP phosphorylation in patient-derived organoid models. Here, we analyzed 45 colon cancer organoids (CCOs) selected in the related research article to investigate the role of β-arrestin 2 in YAP phosphorylation. Hematoxylin and eosin (H&E) staining and immunohistochemistry data showed that the CCOs maintained tissue architecture and histological features of their original cancer tissues. Moreover, mutation data detected from RNA-seq (RNA-sequencing) analysis showed that these CCOs retained the genetic features of their original colon cancer tissues as well. We also confirmed at the protein level that organoids expressing β-arrestin 2 showed high expression of phosphorylated YAP. These organoid model studies strongly support the related research article that β-arrestin 2 suppresses the activation of YAP in colon cancer.

## Specifications Table

**Table:** 

Subject	Cell biology
Specific subject area	Biological data of colon cancer organoid
Type of data	Imaging, genomic, and protein data supporting the related research article. The data is presented in the form of figures and a table.
How the data were acquired	Microscope, survey, H&E staining, immunohistochemistry staining. Whole transcriptome sequencing data were generated by Illumina sequencing platforms.
Data format	Raw Analyzed
Description of data collection	Raw RNA-seq data were analyzed and quantified gene expression using the TCGA RNA-seq Pipeline (v2) after sequencing quality assurance. Reads that passed the quality check were mapped to the human reference genome (hg19) using MapSplice v2.2.1 [1]. RSEM v1.3.0 [2] was used to transcript quantification and normalized within samples to a fixed upper quartile.
Data source location	Institution: Asan Medical Center City/Town/Region: Seoul Country: Republic of Korea
Data accessibility	Repository name: Data on the evaluation of the relation between β-arrestin 2 and YAP phosphorylation in patient-derived colon cancer organoids Data identification number: DOI 10.17632/5k6d5s7gsx.1 Direct URL to data: https://data.mendeley.com/datasets/5k6d5s7gsx; http://dx.doi.org/10.17632/5k6d5s7gsx.1; https://dx.doi.org/10.17632/5k6d5s7gsx.1; https://data.mendeley.com/datasets/5k6d5s7gsx/1
Related research article	For an article which has been accepted and is in press: M Kim, JM Kim, EJ Cho, CO Sung, J Kim, SJ Jang. β-arrestin 2 suppresses the activation of YAP by promoting LATS kinase activity. *Genes Dis 2021*

## Value of the Data

• The data presented here show the suppressive role of β-arrestin 2 in YAP activation in colon cancers. This data provides accurate evidence obtained using patient-derived models to prove the molecular mechanism that β-arrestin 2 is associated with YAP phosphorylation in colon cancers.
• The data contains results showing the similarity between organoid models and their original cancer tissues. Accordingly, this article may be useful for researchers searching for preclinical models that can faithfully recapitulate the characteristics of the original patient cancer tissues.
• As YAP/TAZ is a key oncoprotein of the Hippo pathway in the nucleus of various tumor cells [3], inhibition of nuclear YAP/TAZ is being highlighted as a potential therapeutic target [4]. The data may provide novel insights into targeting the Hippo pathway of colon cancers through β-arrestin 2 instead of YAP.

## 1 Data Description

**Fig. 1:**
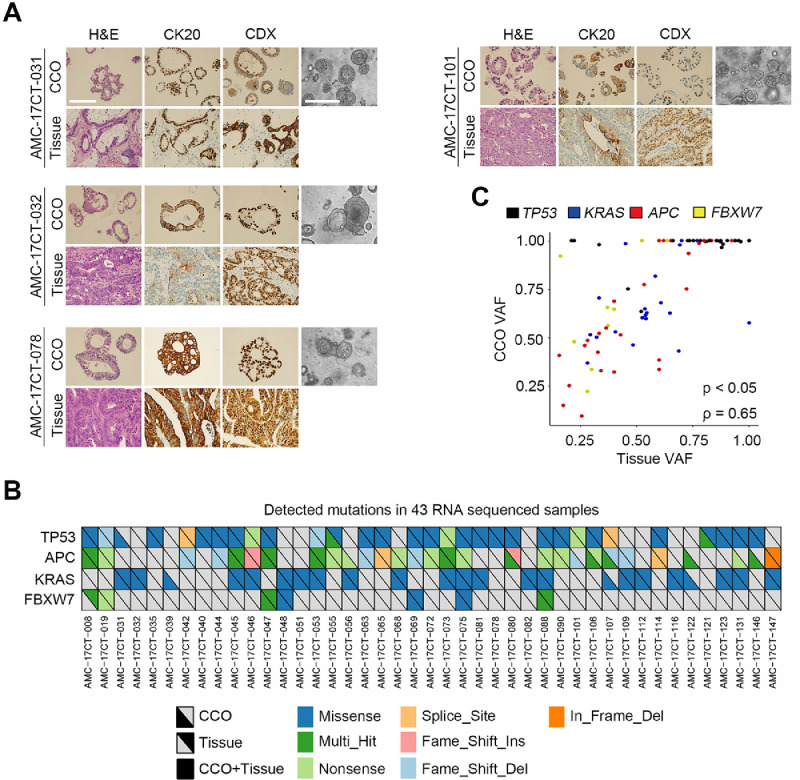
Colon cancer organoids (CCOs) recapitulate the histological and genetic characters of colon cancer tissues. (A) H&E-, IHC-stained and bright-field microscopy images of 4 CCOs and their original colon cancer tissues (Tissue). Scale bars, 200 μm. (B) An oncoprint plot showing major somatic mutations (*TP53, KRAS, APC,* and *FBXW7*) that were observed in 43 CCOs and the matched tissues (The raw data uploaded in Mendeley Data, V1, doi: 10.17632/5k6d5s7gsx.1). (C) Spearman's correlation coefficients of the VAF of the somatic mutations detected in 43 CCOs and the matched tissues (Spearman's correlation test; *ρ* = 0.65, *p* < 0.05). The dots indicate each sample having the indicated mutant gene (*TP53*; black, *KRAS*; blue, *APC*; red. *FBXW7*; yellow).

The data presented in this article demonstrate that β-arrestin 2 promotes YAP phosphorylation in patient-derived colon cancer organoids (CCOs) to support the data in the associated research article by Kim et al. These 45 organoids in this article were selected from 90 CCOs analyzed to the RNA-sequencing in the associated research article by Kim et al. These CCOs were generated from 45 colon cancer tissues including adenocarcinoma and tubular adenoma (Table 1). First, we verified the similarity between CCOs and their matched cancer tissues. Hematoxylin and eosin (H&E) staining and immunohistochemistry (IHC) analyzes showed that our CCOs mimicked the tissue architecture of their original cancers and retained the expression of colorectal adenocarcinoma markers [5] cytokeratin 20 (CK20) and caudal-type homeobox 2 (CDX2) (Fig. 1A). Next, we compared the similarity of genomic features between the CCOs and their original cancer tissues using 43 CCOs that had matched tissues, as the original patient tissues of 2 CCOs did not have the quality for this analysis. We investigated somatic mutations in *TP53, KRAS, APC,* and *FBXW7* genes, which are frequently found in colon cancers [6], and most somatic mutations in tissues were detected in their matched CCOs, as the concordance of somatic mutations ranged from 72% to 90% for the 43 samples (Fig. 1B). In addition, we calculated the variant allele fraction (VAF) distribution to examine the correlation between tissues and CCOs on these 4 genes. As expected, the VAF values of these mutations in CCOs were correlated with those in tissues (Fig. 1C; *ρ* = 0.65, *p* < 0.05). Moreover, the VAF values in CCOs and tissues had a wide range from 0.1 to 1.0 (Fig. 1C), indicating that CCOs show genetic heterogeneity with various subclonal cancer cell populations as in tissues. However, the VAF values of CCOs were generally higher than those of original tissues, suggesting that CCOs were generated from pure cancer cells without non-tumor cells such as stromal and normal cells [7]*.* In conclusion, CCOs that we generated represent the cancer characteristics of their original tissues.

**Table 1:** Detailed information on the 45 patient-derived colon cancer organoids (CCOs) used in this article.

**Fig. 2:**
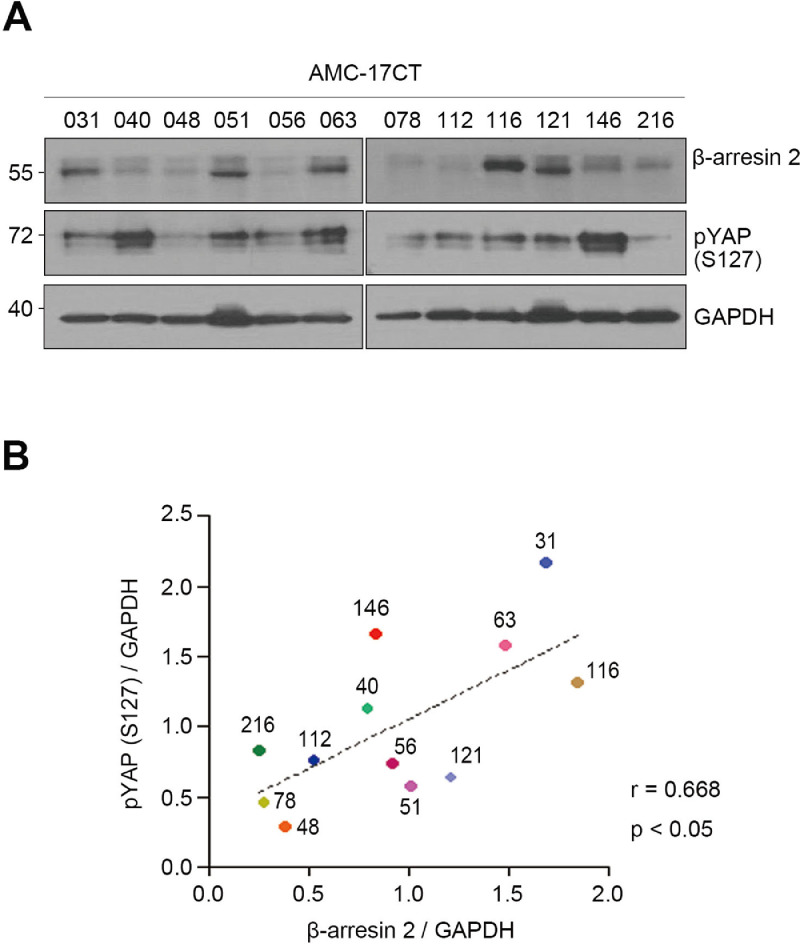
β-arrestin 2 expression has a positive correlation with phosphorylated YAP in CCOs. (A) Western blotting analysis of β-arrestin 2 and pYAP in 12 CCOs (B) Regression coefficients of β-arrestin 2 and pYAP using the Western Blotting results of 12 CCOs in (A) (Linear regression test; *r* = 0.668, *p* < 0.05). β-arrestin 2 and pYAP expression levels were normalized to that of GAPDH. The dots indicate each CCO and are marked with their respective identification number (“AMC-17CT-“ omitted).

Based on the expression of β-arrestin 2 and YAP in the RNA sequencing data (uploaded in Mendeley Data, V1, doi: 10.17632/5k6d5s7gsx.1), we divided the CCOs into the AY1 group (low β-arrestin 2/high YAP expression), AY2 group (high β-arrestin 2/low YAP expression), or the group that did not have a statistical significance about the correlation between β-arrestin 2 and YAP. The criteria for this grouping are explained in detail in the associated research article by Kim et al. Of the 45 CCOs, 18 CCOs were grouped into the AY1 group, 20 in the AY2, and 7 CCOs did not belong to any groups (non-group) (Table 1). Next, to demonstrate the role of β-arrestin 2 in YAP activation, we investigated the correlation between β-arrestin 2 and phosphorylated YAP (pYAP) at the protein level; specifically, we focused on the phosphorylation at Ser 127 of YAP (pYAP S127), which is the form that blocks YAP transcriptional activity [8]. For Western Blotting analysis, we randomly selected 12 of 45 CCOs belonging to the AY1, AY2, or non-group (Fig. 2A). As a result, β-arrestin 2 and pYAP showed a positive correlation in CCOs (Fig. 2B; *r* = 0.668, *p* < 0.05). This result is consistent with our RNA expression data in the associated research article by Kim et al. that β-arrestin 2 shows a reverse correlation with YAP.

Collectively, our dataset shows that our CCOs maintained the nature of their original cancer tissues and that the CCOs were critically useful in investigating the role of β-arrestin 2 in YAP activation. Especially, CCOs with a strong expression of β-arrestin 2 were more likely to show the phosphorylation of YAP than other CCOs.

## 2 Experimental Design, Materials and Methods

Tissue preparation and culture of CCOs. The samples were surgically resected from colon cancer patients with patients’ consent and directly transported to the laboratory on ice within 1 h in cold Hank's balanced salt solution (HBSS) with antibiotics (Lonza, Basel, Switzerland). Samples were sectioned into approximately 1–2 mm^3^-sized pieces by sterile blades after washing three times with cold HBSS. The sectioned tissue samples were dissociated by incubating with 0.2 U/μL collagenase II (Gibco, Carlsbad, CA, USA), 1% penicillin/streptomycin (Gibco), and 0.5 mg/mL amphotericin B (2% antibiotics, Sigma, St Louis, MO) in DMEM/F12 medium (Lonza) at 37 °C for 40–90 min with intermittent agitation. The suspensions were centrifuged at 161 rcf for 5 min and washed with DPBS (Welgene, Seoul, Republic of Korea). After washing, the suspensions were repeatedly triturated by pipetting and passed through 100 μm cell strainers (BD Falcon, CA, USA). The strained cells were centrifuged at 40 rcf for 3 min, and the pellet was resuspended in 100 μL minimum basal medium for colorectal cancer organoid (CCO MBM) containing a serum-free medium (DMEM/F12; Gibco) supplemented with 50 ng/mL human epidermal growth factor (Invitrogen, Waltham, MA), B27 (Invitrogen), 1 mM n-acetylcysteine (PeproTech, NJ, USA), 10 mM nicotinamide (PeproTech), 10 nM gastrin I (PeproTech), 500 nM A83–01 (PeproTech), 10 μM ROCK inhibitor (PeproTech), and 1% penicillin/streptomycin (Gibco).

To establish organoids, the resulting cell suspensions with 200 μL Matrigel (Corning, NY, USA) were allowed to solidify on 2 wells of pre-warmed 6-well culture plates (Corning) at 37 °C for 10 min. After gelation, 3 mL CCO MBM was added to the wells. The medium was changed every 3–4 days, and the organoids were passaged after 1–3 weeks. For passaging, solidified Matrigel drops containing the organoids were harvested using cold DPBS into a conical tube and centrifuged at 112 rcf for 3 min at 4 °C. The pellet was washed with cold DPBS and centrifuged at 250 rcf for 15 min at 4 °C. The pellet was divided into two layers, which were the Matrigel layer and the cell layer containing organoids. The Matrigel layer was removed from the pellet and organoids were resuspended in 2 mL TrypLE Express (Invitrogen) and incubated for 10 min at 37 °C for dissociation. After incubation, 10 mL of DMEM/F12 containing 10% FBS was added, and the samples were centrifuged at 112 rcf for 3 min. The pellet was washed with DPBS and centrifuged at 112 rcf for 3 min. The pellet was resuspended in CCO MBM + Matrigel (1:3) and reseeded at 1:3 to 1:4 ratios to allow the formation of new CCOs.

Whole-transcriptome sequencing and data processing. To extract RNA from CCOs, we used CCOs (> passage 3) cultured in 4–6 wells of 24-well culture plates (Corning). To obtain the CCOs, solidified Matrigel drops containing the CCOs were harvested in cold DPBS into a conical tube and centrifuged at 112 × g for 3 min at 4 °C. The pellet was washed with cold DPBS and centrifuged at 250 × g for 15 min at 4 °C. Total RNA was extracted from the pellets using the RNeasy Mini Kit (Qiagen, Hilden, Germany) according to the manufacturer's protocol. A cDNA library was constructed using the TruSeq RNA Access Library Prep Kit (Illumina, Inc., CA, USA) and 1 mg of total RNA. All cases passed the cDNA library quality assurance (minimum requirement: > 5 nM). Finally, 100-nt paired-end sequencing was performed using the HiSeq 2500 platform (Illumina, Inc.). Total RNA sequencing was performed using protocols described elsewhere [7].

We analyzed the mutation profiles by using whole-transcriptome data obtained from 43 CCOs and matched tissue samples. To detect mutations from whole-transcriptome data, reads from RNA sequencing were mapped to the human reference genome (National Center for Biotechnology Information build 37) using STAR (2.7.3) [9]. After recalibration of the bases on the RNA-Seq reads, GATK HaplotypeCaller (3.8.0) and GATK Mutect2 (4.0.2) were run to search for mutations in the RNA BAM files. Mutations discovered using HaplotypeCaller were filtered based on the following criteria: FS > 30.0 and QD < 2.0. For variants detected by Mutect2, only the “PASS” from FilterMutectCalls was used [10]. RNA BAM depth at the mutated position was calculated using the “depth” option in samtools.

Hematoxylin and eosin (H&E) staining and immunohistochemistry. We performed H&E staining and immunohistochemistry using protocols described elsewhere [7]. Specifically, all samples containing tissues and matched organoids were prepared to paraffin block by fixing in 4% paraformaldehyde, dehydration, paraffin embedding, sectioning, and standard hematoxylin and eosin (H&E) staining. For immunohistochemical staining, samples were incubated with anti-cytokeratin 20 (CK20; K_s_20.8; 1:400; #GA777; Dako, CA, USA) and anti-CDX2 (D11D10; 1:1000; #12,306; Cell Signaling Technology, MA, USA) antibodies. The sections were subsequently incubated with the corresponding secondary antibodies (1:5000; Vector Laboratories, CA, USA) and visualized using the ultraView Universal DAB Detection kit (Ventana Medical Systems). Nuclei were counterstained with Harris hematoxylin. Images were acquired using the CELENA X System (Logos Biosystems, Anyang, Republic of Korea).

Western blotting. Twelve CCOs out of 45 CCOs (> passage 3) were selected for protein expression analysis. After growing up to 150–200 μm dimeter, the CCOs were harvested from 6 to 12 wells of 24-well culture plates (Corning) and lysed in lysis buffer (Cell Signaling Technology) containing phosphatase inhibitor cocktail C (Santa Cruz Biotechnology, TX, USA). We measured the concentration of proteins in cell lysates using the Enhanced BCA Protein Assay Kit (Pierce Biotechnology, Inc., MA, USA) and 30 μg of proteins were loaded in each lane. Proteins were subjected to SDS-PAGE and transferred to nitrocellulose membranes with a pore size of 0.45 μm (Amersham, GE health care life sciences, PA, USA). The membranes were blocked for 1 h at room temperature with 5% skim milk (BD Difco, NJ, USA) in 1 × Tris-buffered saline Tween-20 (TBST) (25 mM Tris, 150 mM NaCl, 2 mM KCl, pH 7.4, supplemented with 0.1% Tween-20). Then, the membranes were incubated overnight at 4 °C with the following primary antibodies diluted with 5% skim milk in 1 × TBST: anti-β-arrestin 2 (C16D9; 1:1000; #3857; Cell Signaling Technology), anti-phosphorylated YAP (pYAP S127; 1:1000; #4911; Cell Signaling Technology), and anti-glyceraldehyde 3-phosphate dehydrogenase (GAPDH; 1:5000; #sc-32,233; Santa Cruz Biotechnology). Incubation with horseradish peroxidase (HRP)-conjugated goat anti-rabbit or anti-mouse IgG secondary antibodies (1:1000; Enzo Life Sciences, Inc., NY, USA) was performed for 1 h at room temperature.

Statistical analysis. To investigate the similarity of somatic mutation analysis between CCOs and tissues, Spearman's correlation test was performed by using VAF values. To investigate the correlation between β-arrestin 2 and pYAP expression, a linear regression test was performed by using values of protein levels normalized to GAPDH. Statistical analysis was performed using R version 4.0.2 (R Foundation for Statistical Computing, Vienna, Austria) and SPSS Version 24. The graphs were generated using GraphPad Prism 5.0.1. *P* values < 0.05 were considered to indicate statistically significant differences.

## Ethics Statement

Human specimens. This research has been carried out in accordance with The Code of Ethics of the World Medical Association (Declaration of Helsinki). Small sections (approximately 1–4 cm^3^) of colon cancer tissues were obtained from surgically resected colon cancer specimens at the Asan Bio-Resource Center (Seoul, Republic of Korea; Approval No. 2018-25(179)) with the patients’ consent. The research protocol was approved by the Institutional Review Board of Asan Medical Center (Seoul, Republic of Korea; Approval No. 2018-0152). The entire experimental protocol was conducted in compliance with the institutional guidelines. Samples were determined as tumor or normal tissue on the basis of histopathological assessment. The diagnosis of each case was confirmed by the pathologists at Asan Medical Center.

## CRediT authorship contribution statement

**Minsuh Kim:** Conceptualization, Methodology, Investigation, Formal analysis, Validation, Writing – original draft, Writing – review & editing. **Ji Min Kim:** Methodology, Investigation, Formal analysis, Validation. **Eun Jeong Cho:** Methodology, Software, Validation. **Chang Ohk Sung:** Conceptualization, Software, Validation, Writing – original draft, Writing – review & editing. **Joon Kim:** Conceptualization, Writing – original draft, Writing – review & editing. **Se Jin Jang:** Conceptualization, Methodology, Investigation, Formal analysis, Writing – original draft, Writing – review & editing, Supervision.

## Declaration of Competing Interest

The authors declare that they have no known competing financial interests or personal relationships that have or could be perceived to have influenced the work reported in this article.

## Data Availability

Data on the evaluation of the relation between β-arrestin 2 and YAP phosphorylation in patient-derived colon cancer organoids (Original data) (DIB).
